# Editorial: Biology and Pathology of Tumor Viruses in Animals

**DOI:** 10.3389/fvets.2021.797596

**Published:** 2021-11-16

**Authors:** Rui M. Gil da Costa, Ana Lúcia Abreu-Silva

**Affiliations:** ^1^Post-graduate Programme in Adult Health (PPGSAD), Department of Morphology, Federal University of Maranhão (UFMA) and UFMA University Hospital (HUUFMA), São Luís, Brazil; ^2^Centre for the Research and Technology of Agro-Environmental and Biological Sciences (CITAB), Inov4Agro, University of Trás-os-Montes and Alto Douro (UTAD), Vila Real, Portugal; ^3^Molecular Oncology and Viral Pathology Group, Research Center of IPO Porto (CI-IPOP)/RISE@CI-IPOP (Health Research Network), Portuguese Oncology Institute of Porto (IPO Porto)/Porto Comprehensive Cancer Center (Porto.CCC), Porto, Portugal; ^4^LEPABE–Laboratory for Process Engineering, Environment, Biotechnology and Energy, Faculty of Engineering, University of Porto, Porto, Portugal; ^5^Department of Pathology, State University of Maranhão (UEMA), São Luís, Brazil

**Keywords:** cancer, virus, diagnosis, carcinogenesis, cell lines

The causative agents of cancer include numerous biological carcinogens. While carcinogenic parasites and bacteria have been recognized, viruses comprise most of biological carcinogens. Since the discovery of the Rous sarcoma virus over a 100 years ago (1), numerous tumor viruses have been characterized in animal species, where they induce benign and malignant lesions by deregulating complex cellular signaling pathways. The present Research Topic is focused on tumor viruses that affect animals, as well as on their biology, the mechanisms through which they deregulate key cellular functions and the lesions they cause.

Cui et al. contributed a manuscript describing the new avian leukosis virus J strain LH20180301, isolated from broiler breeder chickens in Southern China. The authors described the occurrence of severe and fast-developing lesions at high incidence in association with this viral strain. They also characterized the genomic features of the LH20180301 strain, providing clues concerning its phylogeny. Finally, specific genomic traits were identified that may help explaining the high aggressiveness of this viral strain.

Li et al. developed a new cell line representing a primary canine mammary gland adenocarcinoma. This is a new model for studying canine mammary cancer *in vitro* and *in vivo*, as the cells were shown to develop xenografts in laboratory mice. The authors provided a molecular characterization of their cell-based model, particularly in what concerns key markers like sex hormone receptors and epidermal growth factor receptor 2. This new model was also employed for testing the efficacy of two anti-neoplastic drugs, rapamycin and imatinib, further demonstrating its potential for pre-clinical drug tests. Overall, the new B-CMT cell line should be a useful tool for pre-clinical research into canine mammary cancer.

A new PCR-based method for detecting and quantifying the expression of the E6 oncogene of bovine papillomavirus (BPV) types 2 and 13 was reported by de Alcântara et al.. This Brazilian group developed an innovative assay using real-time PCR to quantify E6 mRNA in urinary bladder samples, potentially infected with those BPV types. The authors validated the assay using conventional PCR and direct sequencing, providing a new tool for scientists interested in BPV and its carcinogenic effects in the urinary bladder.

Finally, a mini-review about the interactions of BPV and chemical carcinogens from bracken fern was contributed by Oliveira et al.. The Portuguese-Brazilian team provided an update on the 24 BPV types currently recognized and on the mechanisms through which bracken toxins and BPV may synergize to induce cancer in cattle.

From the original discoveries of Peyton Rous in the early 1900s to the present Sars-CoV-2 pandemic, the study of animal viruses has major implications for health and science at large. Research on animal tumor viruses remains a field of interest for veterinarians and other health professionals and will continue to deliver insights into basic biology and health-related applications.

## Author Contributions

All authors drafted the manuscript and approved the final version.

## Funding

This work was supported by the Research Center of the Portuguese Oncology Institute of Porto (project no. PI86-CI-IPOP-66-2017); by European Investment Funds by FEDER/COMPETE/POCI - Operational Competitiveness and Internationalization Program, and national funds by FCT - Portuguese Foundation for Science and Technology under projects UID/AGR/04033/2020, UIDB/CVT/00772/2020 and by Base Funding-UIDB/00511/2020 of the Laboratory for Process Engineering, Environment, Biotechnology, and Energy–LEPABE–funded by national funds through the FCT/MCTES (PIDDAC); and Project 2SMART - engineered Smart materials for Smart citizens, with reference NORTE-01-0145-FEDER-000054, supported by Norte Portugal Regional Operational Programme (NORTE 2020), under the PORTUGAL 2020 Partnership Agreement, through the European Regional Development Fund (ERDF).

## Conflict of Interest

The authors declare that the research was conducted in the absence of any commercial or financial relationships that could be construed as a potential conflict of interest.

## Publisher's Note

All claims expressed in this article are solely those of the authors and do not necessarily represent those of their affiliated organizations, or those of the publisher, the editors and the reviewers. Any product that may be evaluated in this article, or claim that may be made by its manufacturer, is not guaranteed or endorsed by the publisher.
